# Sigma receptors and mitochondria-associated ER membranes are converging therapeutic targets for Alzheimer’s disease

**DOI:** 10.3389/fnins.2025.1733659

**Published:** 2025-12-19

**Authors:** Madhura S. Lotlikar, Jacob C. Zellmer, Raja Bhattacharyya

**Affiliations:** Genetics and Aging Research Unit, Mass General Institute for Neurodegenerative Disease, Henry and Allison McCance Center for Brain Health, Department of Neurology, Massachusetts General Hospital, Harvard Medical School, Charlestown, MA, United States

**Keywords:** sigma-1 and -2 receptors, σ1R/σ2R agonists and antagonists, amyotrophic lateral sclerosis, Alzheimer’s diseases, Huntington and Parkinson diseases, mitochondria-associated ER membrane

## Abstract

Alzheimer’s disease (AD) begins decades before clinical symptoms emerge. The “amyloid hypothesis” suggests that amyloid-*β* (Aβ) deposition initiates a cascade of tau hyperphosphorylation, neuroinflammation, and neuronal loss leading to cognitive decline. The recent success of anti-Aβ therapies such as Leqembi in prodromal or mild cognitive impaired patients underscores the importance of early intervention and Aβ clearance. However, safety and cost limitations highlight the need for alternative therapeutic strategies. Small-molecule modulators of Sigma-1 and Sigma-2 receptors (σ1R and σ2R) have emerged as promising candidates for AD treatment. σ1R agonists exhibit neuroprotective and anti-amnestic effects under pathological conditions without affecting normal cognition. Beyond AD, σ1R is implicated in several neurodegenerative diseases including ALS (amyotrophic lateral sclerosis), Parkinson’s, and Huntington’s diseases, stroke, and epilepsy. σ1R plays a key role at mitochondria-associated ER membranes (MAMs)—specialized lipid raft-like domains that form functional membrane contact sites between the endoplasmic reticulum (ER) and mitochondria. *β*-secretase (BACE1), *γ*-secretase, and their substrates APP and palmitoylated APP (palAPP) localize in the MAMs, promoting amyloidogenic Aβ production. MAMs serve as dynamic hubs for inter-organelle communication, calcium signaling, and lipid metabolism. The “MAM hypothesis” proposes that MAM dysregulation drives early AD pathology and persists throughout disease progression, contributing to neurofibrillary tangle formation, calcium imbalance, and neuroinflammation. This review aims to summarize the current understanding of σ1R-mediated regulation of MAMs and its neuroprotective mechanisms, highlighting potential therapeutic opportunities for targeting σ1R in AD and other neurodegenerative disorders.

## Introduction

Alzheimer’s Disease (AD) pathophysiology begins decades before symptoms appear. Translational research, including genetic, biological, and biomarker studies, has significantly advanced our understanding of the “amyloid hypothesis” that proposes that the pathogenesis of is primarily caused by the deposition of amyloid-*β* (Aβ), which triggers tau phosphorylation, neuroinflammation, and neurodegeneration in the brain leading to cognitive impairment and memory deficit (Hampel et al., 2021). The recent success of anti-Aβ therapies (e.g., Leqembi) in patients with prodromal or mild cognitive impairment underscores the importance of Aβ clearance and the value of treating patients before symptoms appear (van Dyck et al., 2023; Budd Haeberlein et al., 2022). However, safety concerns and high costs highlight the need for alternative strategies.

Small-molecule therapies targeting Sigma-1 and -2 Receptors (σ1R and σ2R, respectively), offer a promising approach to treat AD (De Vos et al., 2012; Magalhaes Rebelo et al., 2020). σ1R agonists with favorable pharmacological profiles exhibit anti-amnestic effects in pathological conditions but not normal memory (Magalhaes Rebelo et al., 2020; Weber and Wünsch, 2017; Sałaciak and Pytka, 2022; Uchida et al., 2005; Maruszak et al., 2007; Huang et al., 2011; Feher et al., 2012; Wang and Jia, 2023; Fallica et al., 2021). σ1R is emerging as a unique therapeutic target for several neurodegenerative diseases including Amyotrophic Lateral Sclerosis (ALS), Parkinson’s, Huntington’s, and Alzheimer’s Diseases (PD, HD, and AD, respectively), stroke, and Epilepsy (Piechal et al., 2021; Malar et al., 2023). The precise mechanism underlying the neuroprotective regulatory functions of σ1R ligands also remains unclear.

σ1R is an endoplasmic reticulum (ER)-resident chaperone that under physiological conditions, binds with the chaperone-binding immunoglobulin protein (BiP)/glucose-regulated protein 78 (GRP78) on the ER. Upon activation, σ1R dissociates from BiP/GRP78 and interacts with type 3 inositol 1,4,5-trisphosphate receptor (IP3R3; Weng et al., 2017). IP3R3s then forms a tripartite complex with mitochondrial voltage-dependent anion-selective channel 1 (VDAC1) and glucose-regulated protein 75 (GRP75). This stabilizes the membrane contact sites (MCS) formed between the lipid raft (LR)-like dynamic membrane on the ER and the outer membrane of mitochondria (OMM), biochemically isolated as mitochondria-associated ER membranes or MAMs (Bhattacharyya et al., 2021; Fujimoto and Hayashi, 2011).

MAMs are specialized intracellular sites where β-secretase (BACE1) is found accumulating with its substrates APP (*β*-amyloid precursor protein) or palmitoylated APP (palAPP), along with γ-secretases producing neurotoxic Aβ are found (Bhattacharyya et al., 2021; Pera et al., 2017; Area-Gomez et al., 2009; Bhattacharyya et al., 2013). LR- or MAM-bound palAPP is considered a potential therapeutic target for AD because it serves as a preferential substrate for BACE1, the rate limiting step for Aβ generation *in vitro* and *in vivo* (reviewed in Wlodarczyk et al., 2024). MAMs are dynamic and multifunctional scaffold to enable crosstalk between the ER and mitochondria (Bhattacharyya et al., 2021; Zellmer et al., 2024). Recently coined “MAM hypothesis” proposes that MAMs play a critical role in Aβ production to initiate the pathogenic cascade of AD, including NFT formation, calcium dyshomeostasis, and neuroinflammation (Area-Gomez and Schon, 2017; Schon and Area-Gomez, 2013). In addition to AD, MAM perturbation has also been implicated in ALS, PD, and HD (Prinz et al., 2020; Voeltz et al., 2024).

The purpose of this review is to provide a comprehensive overview of the current understanding of σ1R’s role in neurodegeneration and neuroprotection. We will discuss its molecular mechanisms, interactions with key cellular pathways, and potential therapeutic implications. By elucidating the mechanistic insights into σ1R function, we aim to highlight future directions for research and therapeutic development in neurodegenerative disorders.

## Targeting SIGMA-1 receptor (σ1R) in AD

The Sigma-1 receptor (σ1R), an endoplasmic reticulum (ER)-resident chaperone, is highly expressed in the brain and a promising target for neurodegenerative diseases, including AD (Piechal et al., 2021). σ1R is an atypical type I transmembrane (TM) protein that has no second messenger system (Schmidt et al., 2016). While the cellular stress such as the ER or oxidative stress triggers σ1R function, σ1R has no well-defined endogenous ligands except steroids, such as progesterone, testosterone, and neurosteroids that act as modifiers (Hayashi, 2019; Morales-Lázaro et al., 2019). Most σ1R ligands are synthetic small molecules. Several σ1R ligands show anti-amnestic effects in preclinical models and favorable pharmacokinetics (Sałaciak and Pytka, 2022; Wang and Jia, 2023). Notably, σ1R expression increases in early AD—likely as an adaptive response to cellular stress—and σ1R polymorphisms are linked to AD and other neurodegenerative conditions, and polymorphism in the *σ1R* gene is found to be associated with ALS/FTD (Frontotemporal Dementia), AD, and other neurodegenerative diseases (Uchida et al., 2005; Maruszak et al., 2007; Huang et al., 2011; Feher et al., 2012; Piechal et al., 2021; Luty et al., 2010; Al-Saif et al., 2011; Gregianin et al., 2016; Watanabe et al., 2016; Feher et al., 2012; Jin et al., 2015). σ1R interacts with multiple protein complexes to modulate several signaling pathways such as, cellular calcium homeostasis, excitotoxicity, ER stress, mitochondrial function. These pathways are critical to maintain cellular health and survival. This makes the σ1R an interesting target for the development of drugs for neurological diseases where neuronal loss or degeneration is a key part of the pathology. A postmortem study reported that σ1R levels decreased in hippocampus CA1 region of AD patients (Jansen et al., 1993). Direct visualization of σ1R in live mouse brains using a novel (^11^C)-labeled positron emission tomography (PET) probe, [^11^C]CNY-01, combined with immunocytochemistry (ICC) showed reduced σ1R levels in 5XFAD mouse brains compared to the WT, with σ1R reduction linked to amyloid and neuroinflammation pathologies (Bai et al., 2025). An earlier study using the PET probe [^11^C]SA4503 for σ1R in human brains of patients with early AD found diminished expression levels of σ1R compared to cognitively normal controls (Venkataraman et al., 2022). These findings add to the weight of evidence that the σ1R is important and that σ1R ligands may be of benefit to AD patients at their early stages. Regarding therapeutic potential, many σ1R agonists are FDA-approved and show promising anti-amnestic effects in pre-clinical studies of mild-to-moderate AD (Sałaciak and Pytka, 2022; Uchida et al., 2005; Maruszak et al., 2007; Huang et al., 2011; Feher et al., 2012; Wang and Jia, 2023; Rountree et al., 2013; Hampel et al., 2020). Recently, ANAVEX2-73 (blarcamesine), a mixed sigma-1 and muscarinic receptor ligand, has reached phase 2b/3 trials for AD (Hampel et al., 2020; Macfarlane et al., 2025). σ1R-agonists are gaining attention because they act as anti-amnestic agents only in pathological conditions but not in normal memory (reviewed in Sałaciak and Pytka, 2022).

While σ1R agonists show anti-amnestic effects, several prototypic σ1R agonists including PRE-084, (+)-pentazocine, (+)-SKF10047, 4-(N-benzylpiperidin-4-yl)-4-iodobenzamide (4-IBP), and SA4503 are reported to promote cancer cell proliferation and tumor growth, while some of these putative agonists inhibit cell proliferation and trigger cell cycle arrest and a few appear to have no effect on cell proliferation and tumor growth (Megalizzi et al., 2009; Megalizzi et al., 2007; Kim and Maher, 2017). These contradictory findings may be because the notion of agonist and antagonist classifications may be misleading for σ1R because it is an atypical type I transmembrane (TM) protein that has no second messenger system (Schmidt et al., 2016). σ1R agonists and antagonists are classified based on their impact on σ1R’s binding affinities with its partners, such as BiP/GRP78, which often makes their classification misleading (Hayashi and Su, 2007; Kim and Bezprozvanny, 2023). The ability to potentiate neurite outgrowth in PC12 cells, and the Hill slope factor of their binding isotherms, with a slope ≈ 1 indicate an agonist. A shallow slope factor (slope ≈ 0.5) indicates an antagonist (Malar et al., 2023). Despite these methods to classify agonists and antagonist, σ1R has a single binding pocket for all ligands (agonists or antagonists; Sałaciak and Pytka, 2022), which causes the available functional assays to lack selectivity in identifying σ1R agonists or antagonists, generating further ambiguity in the classification. In conclusion, the absence of identified intrinsic activity and promiscuous binding pocket, the concept of σ1R “agonism” and “antagonism” is atypical. Therefore, the term modulator may more accurately define compounds with affinity for σ1R (Kim and Maher, 2017; Su et al., 2010). Even in the absence of a classic second messenger, the “biased signaling” may provide a framework to reconcile the seemingly contradictory effects of σ1R agonists and antagonists on MAM stability and disease outcomes. A ligand’s effect may also depend on its “functional selectivity” toward its protein partners (e.g., BiP, IP3R, GRP75 etc.) and either stabilizes or destabilizes based on the cellular context. Different ligands may also stabilize distinct σ1R conformations, each preferentially engaging in specific downstream pathways at the MAM interface. Thus, ligand-dependent conformations can yield context-specific effects rather than simple “on/off” responses. Recognizing and exploiting this biased signaling offers an opportunity to design stage-specific σ1R modulators for neurodegenerative disease intervention. Testing ligands in early vs. late disease animal models could reveal whether agonist actions help acutely and antagonist actions help chronically, or vice versa.

The lack of known intrinsic activity of σ1R suggests that targeting σ1R will have limited off-target effects. This makes σ1R ligands valuable small molecule therapeutics to treat neurological diseases including AD. σ1R ligands exert their actions through allosteric modulation of protein–protein interactions (PPIs) and signaling systems involved in multiple pathophysiological processes, including calcium dyshomeostasis, ER stress, autophagy, excitotoxicity, mitochondrial dysfunction, and reactive oxygen species (ROS) scavenging. Thus, selecting σ1R ligands targeting a specific pathology without impacting physiological functions would require a comprehensive understanding of each ligand or modulator.

## Targeting the SIGMA-2 receptor (σ2R) in AD

Several anti-amnestic σ1R ligands show affinity toward its subtype sigma-2 receptor (σ2R) that has overlapping pharmacological properties. However, despite their similarities in nomenclature and ligand affinities, σ1R and σ2R are not splice variants. These receptors are products of entirely different genes on different chromosomes (Hellewell and Bowen, 1990; Izzo et al., 2014; Yi et al., 2017; Abate et al., 2015; Chu et al., 2015). More precisely, the σ1R is product of a non-opioid intracellular receptor gene located in chromosome 9. σ2R was first identified as the product of Progesterone Membrane Binding Component-1 (*PGRMC1*) gene, but most recent studies have concluded that σ2R gene is a product of Transmembrane 97 (*TMEM97*) gene (Abate et al., 2015; Chu et al., 2015). However, there is a strong possibility that both TMEM97/σ2R and PGRMC1 may be involved in the same biochemical pathways within the cell because both are implicated with cholesterol trafficking and disorder (Riad et al., 2018). Several σ1R and σ2R ligands, such as PRE-084, SA4503, and Rivastigmine, are showing promising anti-amnestic effects in pre-clinical studies of mild-to-moderate AD (Sałaciak and Pytka, 2022; Alhazmi and Albratty, 2022; Rountree et al., 2013).

We reported before that rivastigmine, a United States Food and Drug Administration (FDA)-approved anti-amnestic drug currently under preclinical studies for mild or moderate AD (Alhazmi and Albratty, 2022; Rountree et al., 2013), lowered the frequency of tight MAMs and reduced Aβ generation *in vitro* cellular model of AD (neuro-2A cells constitutively expressing human APP; N2A_APP_) in a dose-dependent manner (Zellmer et al., 2024; Zellmer et al., 2025). This is consistent with several studies demonstrating that rivastigmine treatment lowers Aβ levels and increases neuroprotective sAPPαlevels in cultured neurons and AD mice (3XTg) (Ray et al., 2020; Bailey et al., 2011). Rivastigmine is a cholinesterase inhibitor and is found to restore neuronal plasticity *in vitro* via both σ1R and σ2R (Terada et al., 2018), suggesting rivastigmine as a ligand for both σ1R and σ2R. Whether rivastigmine is an agonist or antagonist for either σ1R or σ2R needs confirmation. Notably, the putative σ1R agonists are found anti-amnestic and neuroprotective, whereas the σ2R antagonists show neuroprotective property, specifically against Aβneurotoxicity. Similarly, while σ1R agonists promote anti-apoptotic signaling, σ2R antagonists block apoptosis. Therefore, despite the lack of homology, the similar binding profile and opposing activities of σ1R- and σ2R-ligands suggest that both receptors need to be considered when designing novel drugs.

σ2R is found enriched in the synapses in AD brains and hiPSC-derived neurons where σ2R colocalizes and interacts with Aβ oligomers at the synapses of AD neurons (Colom-Cadena et al., 2024). CT1812 is one of the allosteric σ2R antagonists developed by Cognitive Therapeutics Inc. (CogTx) for treating AD (Rishton et al., 2021). This drug works by displacing Aβ-oligomer from synaptic σ2R and is the first σ2R antagonist mimicking the protective effects of the Icelandic A^673^T mutation in the APP gene (Harper et al., 2015). CT1812 is currently in phase 1 clinical trials and is relatively safe (NCT03716427 and NCT02907567) in healthy volunteers compared to the placebo-controls (Grundman et al., 2019; Catalano et al., 2017). CT1812 has higher selectivity and affinity for the σ2R and minimal off-target effects (Izzo et al., 2014). Transcriptomic analysis revealed several differentially expressed genes (DEGs) between CT1812-treated and untreated hiPSCs+AD brain homogenates (source of Aβ oligomers; Colom-Cadena et al., 2024). Among the top-most significant transcripts, the cell adhesion Protocadherin gamma-B4 (*PCDHGB4*) and several glia-modulating genes and astrocytic biomarker of inflammation in AD. Pathway analysis found important role for astrocytes in protecting synapses and ultimately cognition, suggesting a role for CT1812 in modulating inflammatory pathways and restores synapse health. More detailed study of each DEGs will help uncover the mechanism of action of anti-amnestic *σ*2R-ligands in AD.

Although σ2R antagonists have demonstrated neuroprotective and anti-apoptotic effects, their direct role in MAM biology remains unexplored. Given the essential involvement of lipid rafts and cholesterol in MAM composition—and the known cholesterol-binding property of σ2R (Jin et al., 2022), a comprehensive investigation into the potential convergence of σ1R and σ2R in regulating MAMs could reveal synergistic mechanisms and lead to more effective therapeutic strategies for neurodegenerative diseases. Notably, Oyer et al. have provided a holistic, lipid-centric perspective on the convergence of both sigma receptors in the context of cancer treatment, offering valuable insights that may extend to neurodegenerative disease mechanisms (Oyer et al., 2023).

To date, there has been no direct evidence of whether σ2R directly influences the architecture or function of mitochondria-associated ER membranes (MAMs). One possibility is that, unlike σ1R, σ2R does not localize to or regulate MAMs. If so, this would identify a distinct downstream pathway through which σ1R and σ2R exert non-overlapping effects. Several lines of evidence suggest that despite their overlapping pharmacological profiles, σ1R and σ2R differ substantially in their biological functions (Schmidt and Kruse, 2019). For example, the two receptors have opposing roles in neuropathic pain and may similarly diverge in regulating cell survival and death (Sánchez-Blázquez et al., 2020). σ1R and σ2R also exhibit complex and partially opposing actions in adipogenesis and obesity—conditions that are rising at an alarming rate in the United States. Deletion of both receptors protects animals from diet-induced adiposity; however, the effects of single-receptor loss are sexually dimorphic. σ1R deletion protects both male and female mice, whereas σ2R deletion protects only males. Moreover, σ2R ablation but not σ₁R loss increases fatty-acid oxidation, indicating mechanistic divergence between the receptors. None of these metabolic effects are observed in female σ2R -knockout mice (Li et al., 2022), further highlighting sexual dimorphism.

Together, these findings underscore that σ1R and σ2R regulate distinct biological processes. Further mechanistic studies will be essential to determine whether their divergence extends to MAM organization or signaling, which could ultimately enable precision therapeutic strategies targeting receptor-specific pathways.

## Targeting the σ1R to modulate mitochondria-associated ER membranes (MAMs) in AD

σ1R activation stabilizes MAMs and facilitates calcium transfer from the ER to mitochondria through the IP3R3-GRP75-VDAC1 channel. GRP78-free σ1R may also translocate from the MAM to other cellular compartments such as the plasma membrane, the ER membrane, and the nuclear envelope (Hayashi and Su, 2007). σ1R then interacts with various cellular interaction partners including ion channels, receptors, and kinases (Su et al., 2010). Furthermore, σ1R can translocate from MAMs to plasma membrane where it directly or indirectly modulates intracellular calcium homeostasis by regulating activities of various plasma membrane elements including N-methyl-D-aspartate receptors (NMDARs), voltage-gated calcium channels (VGCCs), acid-sensing ion channel a (ASIC1a) and stromal interaction molecules 1 (STIM1)/Orai1 complex. ER-stress or reactive oxygen species (ROS) activates σ1R resulting in its dissociation from its cognate co-chaperone BiP and stabilizes IP3R3 to form MAM-stabilizing IP3R3-GRP75-VDAC1 anchoring complex. Cancer cells express active σ1R that plays a crucial role in apoptosis by regulating [Ca^2+^] efflux across the ER and mitochondria after forming a complex with IP3R3 and anti-apoptotic proteins Bcl-2 and Ras-related C3 botulinum toxin substrate 1 (Rac1; Weng et al., 2017; Natsvlishvili et al., 2015). Increase Ca^2+^ signaling from the ER into mitochondria alters cell’s electrical plasticity, allowing the cell to become better suited for survival in a cancerous environment (Hayashi and Su, 2007).

Both MAM stabilization and calcium homeostasis are becoming increasingly relevant in AD pathogenesis. “Calcium hypothesis” is an emerging field suggesting that sustained changes in molecular mechanisms that regulate cellular [Ca^2+^] homeostasis, beyond the normal modulations in the cellular [Ca^2+^] that occur during the typical depolarization-repolarization cycles of a healthy neuron, play a critical role in age-related neurodegeneration, including AD (A.s.A.C.H. Workgroup, 2017). Polymorphism in the *σ1R* gene is found to be associated with ALS/FTD and AD (Uchida et al., 2005; Maruszak et al., 2007; Huang et al., 2011; Feher et al., 2012; Luty et al., 2010; Al-Saif et al., 2011; Gregianin et al., 2016; Watanabe et al., 2016). Genetic silencing of the *σ1R* gene in an ALS mouse model (sigmar1^−/−^) showed a significant reduction of MAMs in the neurons, with greater effects in axons (Bernard-Marissal et al., 2015). The putative σ1R-antagonist NE-100 dramatically reduced MAM levels, primarily in the neuronal processes or axons of a 3-dimensional human neural model of AD (Bhattacharyya et al., 2021). These studies indicate that σ1R is an upstream regulator of MAM.

As a proof-of-concept study, we reported that the highly specific σ1R agonist PRE-084 increased, while the antagonist NE-100 significantly reduced axonal Aβ release from a well-characterized 3-dimensional (3D) neural model of familial AD (FAD) (Bhattacharyya et al., 2021; Zellmer et al., 2024; Zellmer et al., 2025). Recently, we reported that the siRNA-mediated σ1R-knockdown (σ1R-KD) also reduced Aβ release from the 3D FAD model. Consistent with the results, several reports have demonstrated σ1R-antagonist actions mimicking phenotypes observed in genetic knockdown (KD) or knockout (KO) animal models (Kim and Maher, 2017; Maurice and Su, 2009; Merlos et al., 2017). The findings suggest that σ1R antagonist NE-100 may serve as small molecule therapeutics contradicting the consensus that σ1R agonists are anti-amnestic. Although, it is difficult to reconcile the discrepancies, challenges remain in identifying specific σ1R ligands due to their atypical nature lacking a second messenger system, as described above.

## Direct targeting of MAM

Pharmacological inhibitors of MAM-resident SOAT1, e.g., K-604 and F12511, lowered AD pathology by increasing Aβ clearance by upregulating microglial MAMs (Shibuya et al., 2014; Shibuya et al., 2015). K-604 also reversed the microglial Aβ-clearance by reducing the levels of microglial neutral lipids in mice and hiPSCs that are deficient in AD-risk genes *TREM2* or *APOE* (Nugent et al., 2020).

Emerging evidence suggests that in addition to regulating amyloid pathology, MAM structures also play a significant role in modulating synaptic function, which is crucial in exacerbating cognitive impairment in AD (Leung et al., 2021; Ferreira et al., 2015). MAM tethering proteins VAPB (VAMP-associated protein B) and PTPIP51(Protein Tyrosine Phosphatase Interacting Protein 51) interact at the synapses to regulate synaptic function (Gómez-Suaga et al., 2019). Defective protein synthesis in the neurons impacts synaptic plasticity, causing memory impairments in AD (Oliveira et al., 2021; Ma et al., 2013; Ribeiro et al., 2023). Counterbalancing protein synthesis in the neurons or synaptic compartments may be a potential therapeutic strategy for early treatment.

Several FDA-approved, synthetic or natural compounds showing promising results in cancer and metabolic disorders are MAM modulators (Magalhaes Rebelo et al., 2020). However, their effect on neurodegenerative diseases is not well studied. MAM modulators may be categorized into three major classes:

### Class I MAM modulators

These are small-molecule reagents that target MAM structural components, e.g., gap width, length, and localization. A synthetic compound named LDC-3/Dynarrestin is one of the first-generation *Class I* MAM modulator that perturbs the length, gap width, and intracellular distribution of MAMs by directly targeting the mitochondrial protein tyrosine phosphatase-interacting protein 51 (PTPIP51) and disrupting its anchoring with the ER-resident vesicle-associated membrane protein-associated protein B (VAMPB; De Vos et al., 2012).

Axonal dystrophies (AxD) are key therapeutic targets that are marked by neuritic swellings and dystrophy, contributing to neurotoxic Aβ accumulation and memory loss (Leuner et al., 2012; Silva et al., 2012). A disease-related mutation (R95Q) in the MAM-tethering protein, mitofusin 2 (MFN2) disrupts mitochondrial transport and fusion, leading to the development of characteristic features of AxD while having little to no detrimental effect on the cell body (Zhou et al., 2021; Misko et al., 2012). One of the first *Class I* MAM modulators is a group of small-peptide mimetics, also termed mitofusin (MFN) agonists, that mimic the intramolecular interacting domains of the mitochondrial mitofusin 2 (MFN2), thereby restoring mitochondrial dynamics in neurodegenerative Charcot–Marie-Tooth type 2A disease (CMT2A) (Rocha et al., 2018). MFN2 is a well-studied MAM anchoring protein that is primarily located in the mitochondria in “closed” or “open” forms guiding mitochondrial fusion and fission, respectively. MFN2 [1–751 amino acid (aa)] forms an inactive “closed” conformation via an intramolecular interaction between the heptad repeat (HR1) domain (aa 338–418), residing between the active (GTPase) and transmembrane (TM) domains, and the C-terminal HR2 domain (aa 681–751). The “closed” conformation leads to mitochondrial fragmentation, while the “open” conformation favors mitochondrial fusion. CMT2A is the prototypical neuronal disorder of defective mitochondrial fusion and impaired mitochondrial trafficking leading to mitochondrial fragmentation and axonal degeneration. Competing peptide mimetics, e.a., Cpd A (compound A) and Cpd B (compound B), analogous to the interactive area within the MFN2 HR1 domain disrupted HR1-HR2 interaction in CMT2A neurons carrying inactive MFN2 mutations. The mimetics allosterically activated MFN2 by disrupting the HR1-HR2 interaction and converting the inactive “closed” conformation to active “open” conformation leading to the reversal of mitochondrial dysmotility, fragmentation, depolarization, and fusion. Whether the mechanism of action of these mimetics involves MAM modulation remains largely unknow.

Although MFN2 is primarily mitochondrial, approximately 5–10% is localized to the ER, where it contributes to MAM formation and function. Anchors (de Brito and Scorrano, 2008). Despite no direct association of the MFN2 gene with AD, disease-related mutation (R^95^Q) in MFN2 disrupted mitochondrial transport and fusion, leading to the development of characteristic features of axonal dystrophy (AxD), a key therapeutic target marked by neuritic swellings that contribute to neurotoxic Aβ accumulation and memory loss with little to no detrimental effect on the cell body (Leuner et al., 2012; Silva et al., 2012; Zhou et al., 2021; Misko et al., 2012). However, targeting MFN2 to modulate MAM formation is challenging because knocking out *MFN2 in vitro* or *in vivo* resulted in reduction of MAM formation in some studies and increase in other studies (Casellas-Díaz et al., 2021; Sugiura et al., 2013; Filadi et al., 2015; Leal et al., 2016). Therefore, whether MFN2 facilitates or impedes MAM formation remains controversial. Recent discoveries indicate that the mitochondrial MFN2 (MFN2_MT_) in its “open” form not only establish a homotypic contact with the 5–10% MFN2 located in the ER (MFN2_ER_), but also form heterotypic contacts with its isoform MFN1 found exclusively in the ER (de Brito and Scorrano, 2008). Thus, peptide mimetics targeting either the homotypic trans-dimer (MFN2_MT_-MFN2_ER_) or heterotypic trans-dimer (MFN2_MT_-MFN1) may have potential therapeutic properties for several neurological disorders including AD, PD, and ALS, wherein MAM structure and function are contributing factors (Bhattacharyya et al., 2021; Zellmer et al., 2024; Bernard-Marissal et al., 2015; Area-Gomez et al., 2012; Dematteis et al., 2024). Peptide mimetics are emerging as promising therapies for many neurological diseases. For example, a 24-aa peptide (p5) targeting the pathogenic Cdk5-p25 complex with remarkable specificity reduced Tau hyperphosphorylation, Aβ plaques, and improved memory and motor function in mice, without affecting the physiological Cdk5-p35 complex (Pao et al., 2023).

### Class II MAM modulators

These are synthetic or natural small-molecule agents that target MAM-anchoring proteins at their transcriptional, translational, or post-translational level. Metformin is an antidiabetic drug, but long-term use is associated with a lower risk of cognitive decline among dementia patients via mechanisms unrelated to diabetes (Campbell et al., 2018). Although the results in AD are mixed, metformin reduced the risk of all types of dementia including AD if it was administered before dementia began (Ji et al., 2022). 500 mg/day administered to individuals with preclinical or mild cognitive impairment (MCI), or genetically predisposed (eg., APOEε4 homozygotes, or pathogenic SORL1) potentially modify disease progression, making it a promising candidate for repurposing in the prevention of AD. Metformin is in Alzheimer’s Dementia Prevention study (NCT04098666)—is investigating the protective effects of doses up to 2000 mg/day, with results expected in 2027. The mechanism of action of metformin is not known. However, metformin increased the expression levels of MAM-proteins VDAC1, PACS-2, and MFN2 (Guo et al., 2024; Wang et al., 2024; Foretz et al., 2014; Sanchez-Rangel and Inzucchi, 2017). Thus, metformin is a Class II MAM modulator that can be repurposed for AD (Cho et al., 2024; Daly and Imbimbo, 2025). Notably, metformin use was also associated with a significantly increased AD risk, specifically for patients with prolonged diabetes or depression (Ha et al., 2021). Therefore, a comprehensive study of metformin’s role in regulating MAM’s structural (e.g., length and gap width) and functional (e.g., Aβ-generation or clearance, lipid or calcium homeostasis, mitochondrial mobility) properties will be valuable in identifying the dose and duration of metformin treatment to prevent or lower AD risk.

In addition to metformin, several chemotherapeutics are known to either directly or indirectly modulate MAM structure/function and homeostasis. Doxorubicin, for example, is an anti-cancer drug, widely used to treat leukemias, lymphomas, and solid tumors but may develop devastating cardiomyopathy associated with reduced expression levels of the MAM anchoring protein MFN2 (Cagel et al., 2017; Tang et al., 2017). Doxorubicin can also decrease the expression of the anti-apoptotic Bcl 2, enhancing cell death stimuli in breast cancer cell lines (Pilco-Ferreto and Calaf, 2016).

Cisplatin (CIS) is a chemotherapeutic agent potentially functioning as a MAM modulator because its anti-cancer effect is mediated by IP3R (Tsunoda et al., 2005). Moreover, CIS is in a complex feedback mechanism involving the downregulation of MFN2 and GRP75 (Xie et al., 2016). Thus, CIS may be repurposed to treat neurological diseases with MAM pathology. However, the biggest obstacle in repurposing is that the anti-cancer dose of CIS develops CIS resistance and cause serious damage to the brain (Cicek et al., 2024). A comprehensive study of CIS’s impact on MAM architecture (“tightening” or “loosening” effects) may uncover avenues to repurpose this drug without the major side effects.

A recent study has identified the antiepileptic drug clemastine as a potential candidate for repurposing in other neurological disorders (Badawi et al., 2024), including AD, given that epilepsy pathophysiology is strongly influenced by elevated Aβ expression levels (Hickman et al., 2023). Clemastine has previously been shown to prevent cognitive dysfunction in preclinical models of multiple sclerosis and cerebral ischemia (Sałaciak and Pytka, 2022). Mechanistically, clemastine helps maintain cellular homeostasis and prevent cell death through activation of σ1R. Activation of σ1R influences multiple pathological pathways, including calcium homeostasis, ER stress, autophagy, excitotoxicity, mitochondrial dysfunction, and reactive oxygen species (ROS) clearance, thereby exerting broad neuroprotective effects against neuroinflammation, neuronal excitotoxicity and apoptosis (Ruiz-Cantero et al., 2021).

### Class III MAM modulators

These targets upstream regulator(s) of MAM stabilization. σ1R is one of the first upstream regulators of MAM’s structure and function. As described above, numerous studies have established that the σ1R is one of the major therapeutic targets against AD (Piechal et al., 2021), and found that σ1R regulates amyloid pathology by modulating MAMs’ plasticity, specifically their lengths, gap widths, somal vs. axonal distribution, and axonal mobility (Bhattacharyya et al., 2021; Zellmer et al., 2024; Zellmer et al., 2025). The impacts of the putative σ1R-antagonist NE-100 and σ1R/σ2R-ligand rivastigmine on Aβ levels in a 3D neural model of AD describes a novel σ1R/σ2R-MAM axis in regulating amyloid pathology in AD (Bhattacharyya et al., 2021; Zellmer et al., 2024). The innovative method of measuring mitochondrial axonal velocity as a useful mean to quantify MAM gap width stabilization has provided high precision in identifying modulators of σ1R/σ2R-MAM axis to delay or prevent AD pathogenesis. Numerous pharmaceutical drugs and small molecules exhibiting diverse chemical structures and therapeutic/pharmacological profiles act as σ1R or σ2R ligands (Weber and Wünsch, 2017; Fallica et al., 2021). Many σ1R/σ2R ligands (agonists/antagonists/modulators) have been FDA-approved, which could be repurposed for the treatment of AD and ADRDs (Table 1).

**Table 1:** Small-molecule σ1R/σ2R modulators: investigational therapeutics for AD and AD-related disorders (ADRD).

Name	Clinical Status	Company	Target
Blarcamesine	Phase 3*	ANAVEX	S1R, M1R
Rivastigmine	Approved*	Exelon	S1R ≈ S2R
PRE-084	Unknown**	---	S1R>>>S2R
NE-100	Unknown**	---	S1R>>>S2R
CME398	Phase 1***	CogTx	S1R>>>S2R

*, AD; ** anti-amnestic; *** anti-epileptic (Piechal et al., 2021; Malar et al., 2023; Dematteis et al., 2024).

## MAM gap width is a potential therapeutic target

The architecture of MAMs—particularly the gap width between the ER and mitochondria, which ranges from 6 to 80 nm—plays a crucial role in regulating their function (Giacomello and Pellegrini, 2016; Sukhorukov et al., 2022; Zhang et al., 2021; Cieri et al., 2018; Carpio et al., 2021; Csordas et al., 2006; Ziegler et al., 2021). Transmission electron microscopy (TEM) studies reveal that smooth ER (SER) typically forms “tight” MAMs with 8–15 nm gaps, while rough ER (RER) forms wider contacts of 20–30 nm; in some contexts, the gap can reach up to 80 nm (Filadi et al., 2015; Hirabayashi et al., 2017; Kowaltowski et al., 2019). Notably, axonal ER is predominantly SER, and therefore primarily forms tight MAMs (Öztürk et al., 2020).

**Figure 1:**
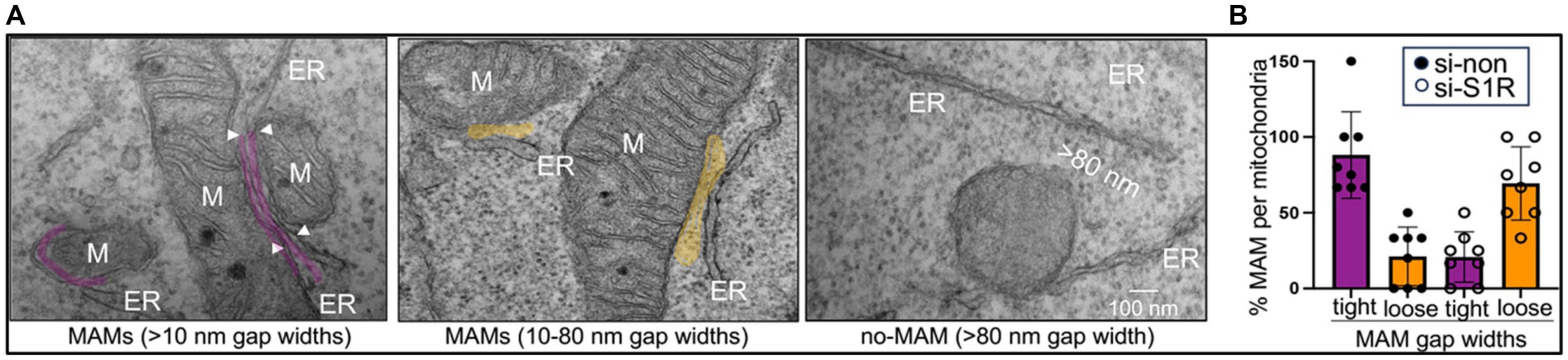
σ1R knockdown decreased the frequency of tight MAMs and lowered Aβ generation *in vitro*. **(A)** TEM images showing ER (ER)-mitochondria (M) contact sites or MAMs of gap widths < 10 nm (purple) or > 10 nm (orange) in ReN-GA cells electroporated with scrambled siRNA (si-non) or siRNA against σ1R (si-σ1R) for 48 h. Mitochondria separated by > 80 nm from the ER are non-MAMs. **(B)** Quantitation of the tight (< 10 nm) or loose (> 10 nm) MAMs in control (si-non) and σ1R-silenced (si-σ1R) cells per mitochondria per frame (MAM per mitochondria). More than seven frames were used for each analysis. An average of 3–6 mitochondria forming tight or loose MAMs from each frame were manually counted (unbiased, *p < 0.05; **p < 0.001; Bhattacharyya et al., 2021; Zellmer et al., 2024).

**Figure 2:**
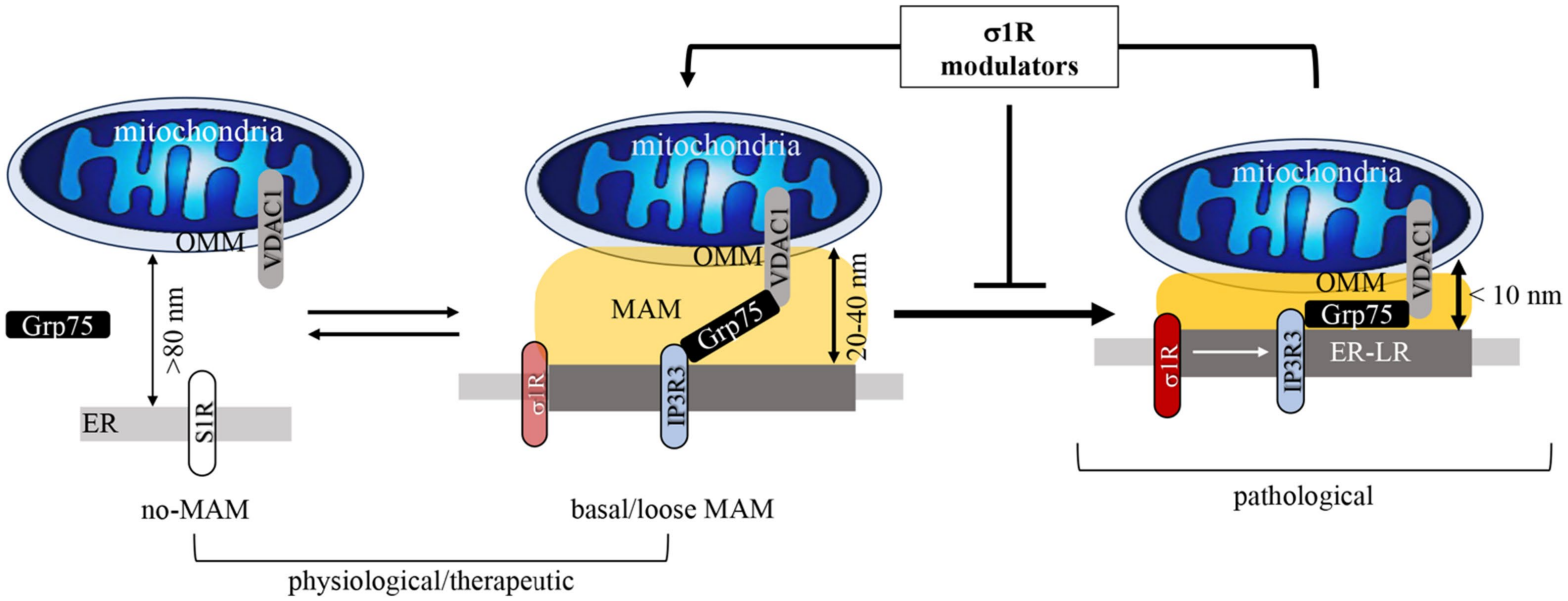
Schematic of σ1R modulators “loosening” the pathogenic tight MAMs to basal or therapeutic loose MAMs. The mitochondria and ER form MAMs (~20 nm gap width) via σ1R-mediated formation of the IP3R3-Grp75-VDAC1 anchor. Abnormal or un-inhibited σ1R results in “tightening” the MAM gap width (<10 nm) and stabilizing tight MAMs that are pathogenic (Aβ-increasing). σ1R modulators reverses the pathogenic tight MAMs to basal or therapeutic MAMs of gap widths ranging from 20 to 40 nm.

MAM gap width defines MAM structure and function. Tight MAMs (~10 nm) are pro-apoptotic and amyloidogenic, whereas medium (~25 nm) or loose (25–80 nm) MAMs appear anti-apoptotic and anti-amyloidogenic (Zellmer et al., 2024; Prudent et al., 2015). In AD, both the structure and function of MAMs, such as their horizontal length or vertical gap width and Aβ generation are perturbed in AD (Figure 1; Schon and Area-Gomez, 2013; Erpapazoglou et al., 2017; Leal et al., 2020). Increased MAM formation has been detected in fibroblasts and post-mortem brain tissue from familial and sporadic AD patients, and AD-associated proteins (APP, BACE1, and *γ*-secretase components) are enriched at these sites patients (Pera et al., 2017; Area-Gomez et al., 2009; Area-Gomez et al., 2012). Our previous work showed that σ1R knockdown reduced tight MAMs (<10 nm) and increased loose MAMs (~2.5-fold), mirroring findings in AD transgenic neurons (Zellmer et al., 2024; Martino Adami et al., 2019). Using optic nerves from mouse and human models—purely axonal systems—we found a > 4-fold increase in tight MAMs in AD axons relative to controls (Zellmer et al., 2024). Using mouse and human optic nerves, reliable *in vivo* models of pure axons, we observed a > 4-fold increase in tight MAMs in AD axons compared to controls (Zellmer et al., 2024). These findings highlight MAM architecture as a potential therapeutic target (Figure 2).

Pharmacological and natural compounds can “tighten” or “loosen” MAMs, and several are currently in preclinical or clinical trials for metabolic disorders and cancer (Magalhaes Rebelo et al., 2020). We showed that disrupting MAM gap width by knocking down the σ1R, introducing constitutive linkers designed to stabilize MAMs of different gap widths, or inducible MAM-tightening biosensors exacerbated Aβ accumulation in a human stem cell-derived neural progenitor ReN cell model overexpressing familial AD-mutant of Aβ-precursor protein, APP^Swe/Lon^ (ReN GA cell-derived neural culture in 3D; Bhattacharyya et al., 2021; Zellmer et al., 2024). Moreover, tightly fused ER–mitochondrial contacts significantly (*p* < 0.001) impaired both anterograde and retrograde axonal transport of mitochondria, whereas medium MAMs (~25 nm) maintained normal dynamics (Table 2; Zellmer et al., 2024; Zellmer et al., 2025). This observation aligns with reduced mitochondrial trafficking in AD cortical neurons (Dai et al., 2002) and AD cybrid cells (Trimmer and Borland, 2005). Thus, promoting “loose” MAMs could restore mitochondrial motility and shift pathogenic Aβ-producing MAMs toward a more homeostatic state.

**Table 2:** Percent axonal movement (overall, retrograde, anterograde).

Parameter	ReN GA (Zellmer et al., 2024)				ReN (naïve)	ReN GA (3D)	
	Overall (%)	Retrograde (%)	Anterograde (%)	Average speed (mm/s)		Aβ_40_ (pM)	Aβ_42_ (pM)
Mito-RFP	53.82 ± 3.3%	25.78 ± 2.31%	28.04 ± 2.48%	0.66 ± 0.03	0.69 ± 0.07	241.7 ± 26.74	13.77 ± 1.52
MAM 1X	26.6 ± 3.4% ***	12.33 ± 2.5% ***	14.27 ± 2.81% ***	0.3 ± 0.02***	0.43 ± 0.04***	377.2 ± 76.87*	26.62 ± 3.86*
MAM 9X	44.79 ± 2.6% ^ns^	23.99 ± 2.17%^ns^	20.80 ± 1.33%^ns^	0.59 ± 0.02 ^ns^	0.62 ± 0.02 ^ns^	158.8 ± 3.27*	17.01 ± 2.02*
MAM 18X	NA	NA	NA	NA	NA	61.93 ± 4.22**	3.33 ± 0.01**

Average speed of Mito-RFP and MitoMERs (light blue) stabilized by tight (MAM1X) or loose (MAM 9X or 18X) MAMs in Ren GA (dark blue) and naïve ReN in (gray). One-way ANOVA. ***p < 0.0001; *p < 0.05, ns, not significant. Total Aβ (Aβ40 and Aβ42) from 3D differentiated ReN GA neurons expressing the MAM stabilizers compared to neurons expressing Mito-RFP. Paired t test. **p < 0.001.

One of the most innovative aspects of our finding is the development of a novel and reliable system to quantify the degree of stabilization of MAMs of different gap widths, circumventing the limitations of traditional techniques like the transmission electron microscopy (TEM), cryo-TEM, or Scanning Electron Microscopies (SEM). TEM and SEM may detect cellular structures at the nanoscale level but cannot quantify MAM’s the degree of MAM stabilization due to the dynamic nature of MAMs (Cieri et al., 2018; Giacomello and Pellegrini, 2016; Stacchiotti et al., 2018). Thus, we have developed an innovative live-cell system that quantifies MAM stabilization by using mitochondrial axonal velocity as a proxy for MAM gap width. Our approach provides a functional metric to screen for therapeutic σ1R ligands for AD. For instance, σ1R ligands that yield average mitochondrial speeds of 0.6–0.7 μm/s (vs. < 0.3 μm/s) may act as small-molecule therapeutics to lower amyloid pathology.

MAM gap width also plays a significant role in modulating mitochondrial calcium levels [Ca^2+^_MT_] and regulating the programmed cell death by modulating IP3R3 forming a complex with the pro-apoptotic BOK and mitochondrial VDAC1 at the MAM. Tight MAMs, termed “full MAMs” are pro-apoptotic, while loose MAMs, denoted as “defective MAMs” reverse apoptosis (Prudent et al., 2015).

Thus, while a σ1R-ligand exhibiting average mitochondrial axonal speed ~0.7 μm/s may serve as anti-amyloid therapeutic (Table 2; Zellmer et al., 2024), we predict that σ1R-ligands exhibiting average mitochondrial axonal speed ~0.3 μm/s will serve as anti-cancer drug. However, we must highlight that the MAM gap width does not always follow a strictly linear relationship with MAM function. While narrowing the gap from >35 nm to ~20 nm enhanced [Ca^2+^_MT_] levels, further tightening below 10 nm reversed the effect (Katona et al., 2022). Therefore, identifying an optimal “therapeutic gap width” may be key to modulating ER–mitochondrial communication safely. Precisely tuning MAM architecture through σ1R ligands could enable targeted interventions to prevent or delay the onset of neurodegenerative or metabolic diseases. Targeting σ1R, an upstream modulator of MAM is safer and efficacious compared to directly targeting MAMs because of their structural and functional diversities. However, the emerging field of structural systems pharmacology (Duran-Frigola et al., 2013; Berger and Iyengar, 2011), which considers the specific properties of the drug targets and their environment, offers promise in developing effective MAM modulators for therapeutic purposes in the future.

**Figure 3:**
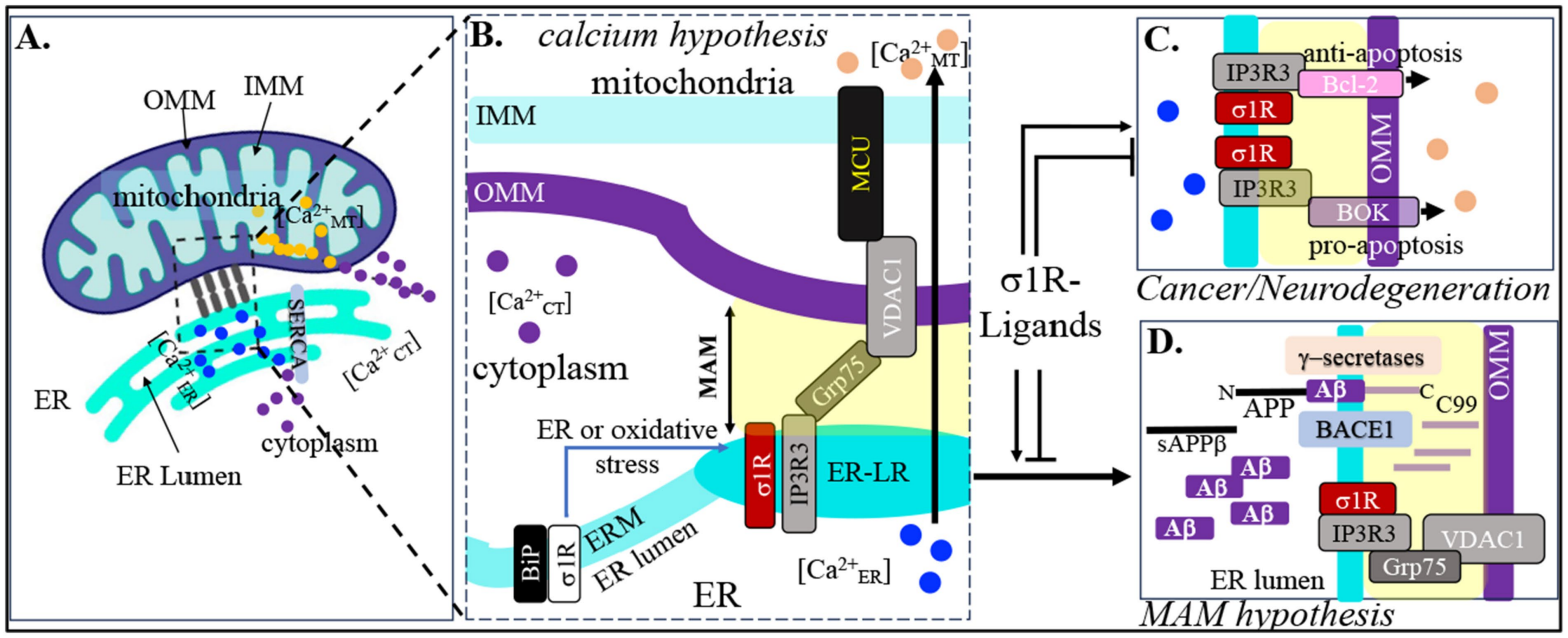
Therapeutic σ1R ligand for amyloidosis or oncogenesis. **(A)** The ER-resident SERCA regulates ER → cytoplasmic calcium transport via Calnexin and TMX1. **(B)** In physiological condition the σ1R remains bound to BiP/GRP78. Upon stimulation (ER- or oxidative-stress) σ1R dissociates from BiP/GRP78 and forms IP3R3-GRP75-VDAC1 anchoring complex to stabilize. VDAC1 then interacts with mitochondrial calcium uniporter (MCU) and promotes ER → mitochondria Ca^2+^-efflux. The “Calcium Hypothesis” of AD asserts that sustained changes in molecular mechanisms that regulate Ca^2+^ homeostasis, play a critical role in several chronic age-related brain disorders, such as AD. **(C)** MAM-tightening/loosening results in increased IP3R3 interaction either with the pro-apoptotic BOK or anti-apoptotic Bcl-2. Inhibiting or enhancing σ1R-stimulation by σ1R-ligands will impact apoptosis, a key molecular pathway that regulates oncogenesis (cancer) or neuronal death (neurodegeneration). **(D)** APP, BACE1, *γ*-secretases as well as C99 and Aβ are all found in MAM fractions. Would increase BACE1-mediated amyloidogenic processing of APP. In contrast, σ1R ligands that maintain “looser” MAM contacts may exert anti-amyloidogenic effects. This aligns with the MAM hypothesis, which posits that MAM perturbation is an early pathological event that remains persistent throughout the disease progression.

A key contributor to AD pathology is the disruption of intracellular calcium (Ca^2+^) homeostasis (Green, 2008). Aβ increases cytosolic Ca^2+^ ([Ca^2+^_CT_]) levels, leading to elevated mitochondrial Ca^2+^ ([Ca^2+^_MT_]), which further promotes Aβ accumulation and neuronal death (Calvo-Rodriguez et al., 2020). Under physiological conditions, the ER maintains Ca^2+^ balance through the sarco/ER Ca^2+^ ATPase isoform SERCA2b, which pumps Ca^2+^ from the cytosol into the ER (Figure 3A) (Britzolaki et al., 2018).

MAM gap width critically regulates Ca^2+^ transport. When MAMs are loose (>35 nm), Ca^2+^ transfer from ER to mitochondria is impaired. Tightening the MAMs to their normal gap width (~20–25 nm) restores physiological Ca^2+^ flux, while excessive tightening (<10 nm) severely reduces mitochondrial Ca^2+^ uptake (Carpio et al., 2021; Katona et al., 2022). Thus, MAM stability modulates ER–mitochondria Ca^2+^ exchange ([Ca^2+^_ER_] ⇌ [Ca^2+^_MT_]) in a non-linear manner. Interestingly, modifying MAMs, either by tightening or loosening the gap widths does not alter cytosolic [Ca^2+^_CT_]. However, the mechanisms driving accelerated mitochondrial Ca^2+^ uptake under pathological conditions remain unclear. Under physiological conditions, the σ1R is bound to BiP/GRP78 in the ER. During ER or oxidative stress, σ1R dissociates and forms an IP3R3–GRP75–VDAC1 complex that tightens MAMs and stabilizes ER–mitochondria communication. VDAC1 then interacts with the mitochondrial calcium uniporter (MCU) to promote Ca^2+^ transfer from the ER to mitochondria ([Ca^2+^_ER_] → [Ca^2+^_MT_]; Figure 3B).

Supporting our “Therapeutic Gap Width” hypothesis, recent evidence in human iPSC-derived astrocytes from PD patients shows that stabilizing the MAM gap width at 20 nm, but not at 10 nm, fully restores mitochondrial Ca^2+^ uptake (Dematteis et al., 2024). This finding directly demonstrates that ER-mitochondria gap width is a critical and tunable determinant of mitochondrial Ca^2+^ uptake and metabolism. This finding is consistent with our proposed model, “tightening” the MAM gap below 20 nm is pathogenic (Aβ-increasing), whereas “loosening” it beyond 25 nm may be therapeutic (Aβ-lowering) (Zellmer et al., 2024; Zellmer et al., 2025). Therefore, normalizing ER-mitochondrial Ca^2+^ transfer or reducing Aβ generation by modulating the MAM gap width may offer a universal therapeutic strategy to restore cellular homeostasis across disorders marked by impaired mitochondrial function or mobility. These findings introduce a new paradigm in which fine-tuning the ER–mitochondria apposition to an optimal gap width could reverse mitochondrial dysfunction, such as dysmotility, fragmentation, depolarization, and defective fusion, implicated in AD (D’Alessandro et al., 2025).

MAM stabilization also influences apoptosis by regulating interactions between IP3R3 and the pro-apoptotic protein BOK or the anti-apoptotic protein Bcl-2 (Figure 3C, upper panel; Carpio et al., 2021). Through these interactions, σ1R-mediated MAM modulation controls programmed cell death—a key pathway linked to oncogenesis.

The major amyloidogenic components, APP, BACE1, γ-secretases as well as the BACE1-cleaved neurotoxic C-terminal 99 amino acid fragment of APP (C99) and γ-secretase-cleaved Aβ are all found in the purified MAM fractions *in vitro* and *in vivo* (Pera et al., 2017; Area-Gomez et al., 2009; Area-Gomez and Schon, 2017). MAM-tightening increased both BACE1-mediated APP cleavage and Aβ generation (Figure 3D, lower panel; Bhattacharyya et al., 2021; Zellmer et al., 2024). Therefore, σ1R ligands that stabilize MAMs at the optimal gap width (20–25 nm) may help maintain physiological Ca^2+^ homeostasis by balancing pro- and anti-apoptotic signaling. Such ligands could have potential therapeutic value as anti-cancer agents by promoting controlled, pro-apoptotic MAM stabilization.

## Future direction

Future research should aim to elucidate the pathway linking MAM modulation to learning and different stages of memory. To date, no studies have directly examined how MAM plasticity (analogous to mitochondrial plasticity (Comyn et al., 2024) in its ability to undergo dynamic changes in gap width, thickness, length, and function) contributes to cellular and network-level neural substrates of learning and memory through σ1R modulation.

We argue that MAMs are critical for regulating the cellular and molecular substrates of brain plasticity underlying various forms of learning, as well as spatial and non-spatial memory. This argument is supported by well-established *in vitro* and *in vivo* evidence showing that modulation of σ1R and mitochondrial plasticity influences memory-related plasticity mechanisms like hippocampal neurogenesis, neuronal spine remodeling, and long-term potentiation and depression (Comyn et al., 2024; Maurice and Goguadze, 2017). Moreover, σ1R interacts with plasticity-related proteins such as cAMP response element-binding protein, c-fos, kinases, and brain-derived neurotrophic factor (BDNF), and modulate both cholinergic and glutamatergic neurotransmission which contributes to memory encoding, consolidation, and retrieval (Maurice and Goguadze, 2017; Crouzier et al., 2020). However, whether this learning-related and learning-induced plasticity is mediated through MAMs remains unknown.

Furthermore, it would be valuable to identify the pathway linking MAM modulation to memory through system-level investigations by integrating *in vivo* electrophysiology and neuroimaging approaches under σ1R modulation and how it relates to cellular plasticity mechanisms. For example, in rodent models, assessing network level plasticity during and after learning by recording regional and simultaneous interregional electrophysiological dynamics (like local field potentials, coherence, and connectivity) within early- AD affected regions like entorhinal cortex, hippocampus and their communication with prefrontal cortex, under regionally localized or systemic σ1R modulation could reveal role of MAM plasticity in behavior. Region-specific delivery of σ1R modulators targeting hippocampal circuits, followed by assessment of electrophysiological correlates such as theta oscillatory power, theta–gamma coupling, and hippocampal–frontal connectivity would be worthwhile.

Furthermore, PET imaging of σ1R in live mouse brains using the [^11^C]CNY-01 probe, as described above, can help identify and quantify the spatial distribution of MAMs across different brain regions under σ1R modulation. Mapping region-specific alterations in pathological MAMs may offer critical insights into the early mechanisms driving AD pathology and help determine whether targeted delivery of σ1R modulators is warranted.

Finally, an investigation into how the environmental and lifestyle factors implicated in AD - such as sleep deprivation, smoking, particulate matter exposure, physical inactivity etc. affect MAM plasticity and whether these changes mediate cognitive deficits in AD could provide valuable insights for preclinical modeling and therapeutic development targeting MAMs. Testing a hypothesis that fear-conditioning learning induces a transient loosening of MAMs in the hippocampus, a process that is disrupted in AD models, can be restored through σ1R modulation. The results of this investigation may connect the established effects of σ1R on mitochondrial function and ER stress, thereby offering a mechanistic link to MAM plasticity.

## Conclusion

The three FDA-approved anti-Aβ immunotherapies (Lequembi, Aduhelm, and Kisunla) lowered AD risks of patients with prodromal or mild AD underscoring the clinical importance of targeting Aβ before symptoms appear (Malar et al., 2023). Additionally, anti-Aβ immunotherapies, e.g., Gamunex, Remternetug, and Sabitnetug have reached Phase 2/3 clinical trials for patients with early AD. The modest efficacy of anti-Aβ immunotherapies in early-stage AD emphasizes that while Aβ remains a relevant target, the real opportunity lies in intervening at the earliest disease stages. The findings are consistent with several preclinical and clinical studies indicating that the therapeutic intervention before symptoms is a more promising approach to prevent or delay AD pathology than targeting after symptoms appear (Hampel et al., 2021; Granzotto and Sensi, 2024). Despite showing promising results, the high cost and significant safety concerns of anti-Aβ immunotherapies highlight the need for safer, cost-effective, and mechanistically distinct alternative therapeutic strategies (van Dyck et al., 2023; Budd Haeberlein et al., 2022).

Synthetic or natural small molecule ligands of σ1R and σ2R are showing promising results in treating neurological disorders (Malar et al., 2023). Given the architectural diversity of MAMs and their critical role in AD pathology, targeting σ1R, an upstream modulator of MAM’s structure offers a novel and potentially safer alternative to traditional anti-Aβ therapies. σ1R appears to be one of the most significant and underexplored therapeutic targets in AD, specifically at the early stages of AD. σ1R’s therapeutic action may involve complex, context-dependent mechanisms targeting either extracellular or axonal Aβ to treat AD at the early stages. The lack of understanding of σ1R-mediated dynamic regulation of MAMs during AD progression and the mechanistic basis for its modulatory effects on Aβ metabolism are critical barriers to therapeutic optimization. Dissecting the intersection of σ1R signaling, ER-mitochondrial communication, and Aβ pathology in AD brains by studying the impacts of σ1R modulation on MAM structure and function will not only advance AD treatment but may also reveal a unifying framework for targeting shared pathogenic pathways across multiple neurological disorders linked to ER-mitochondrial dysfunction.

The focus of this mini review is to examine the convergence between sigma receptors, particularly σ1R, and MAM structure–function as a mechanism underlying disease modification in AD, ALS, PD, and HD. Although interest in the role of sigma receptors in other neurodegenerative diseases is growing, a direct link between sigma receptor activity and MAM structural or functional dynamics has not yet been established. Two recent reviews have highlighted the pleiotropic mechanism of actions of σ1R/σ2R ligands, including modulation of cellular stress and excitotoxicity, which have shown benefits in conditions such as stroke, epilepsy, neuropathic pain, and psychiatric disorders (Piechal et al., 2021; Drewes et al., 2025; Ruiz-Cantero et al., 2024). Together, these findings reinforce the concept that σ1R/σ2R act as master regulators of cellular resilience and represent promising therapeutic targets across a wide spectrum of CNS diseases.
